# Common-onset masking terminates the temporal evolution of orientation repulsion

**DOI:** 10.1167/jov.21.8.5

**Published:** 2021-08-03

**Authors:** Tomoya Nakamura, Ikuya Murakami

**Affiliations:** 1 Department of Psychology, The University of Tokyo, Tokyo, Japan

**Keywords:** appearance, backward masking, object updating, contextual modulation

## Abstract

Our conscious awareness of visual events does not arise instantaneously. Previous studies on backward masking have investigated dynamic internal processes making targets visible or invisible subjectively. However, to understand the whole picture of our rich conscious experiences, the emergence of various phenomenal attributes of consciousness beyond visibility must be delineated. We quantified appearance as the strength of orientation repulsion during common-onset masking and found that masking reduced the repulsion in a near-vertical target grating surrounded by tilted inducers. Furthermore, this reduction was seen only when the inducers were presented together with or after the target. This demonstrates that orientation repulsion involves slow contextual modulation and that masking influences this modulation at a later period. Although appearance was altered as such, orientation discriminability was not reduced by masking in any of our experiments. We propose a process in which internal representations of objects spend a certain amount of time evolving before we become aware of them. Backward masking compulsorily terminates this temporal evolution of internal representations and allows premature representations to arise in our awareness.

## Introduction

### Backward masking as a tool for probing perceptual microgenesis

Backward masking occurs when a brief stimulus (called a target) is made less visible or, in some cases, completely invisible by another stimulus (called a mask) following the target. As there is no difference in inputs between masking and no-masking situations until the mask is delivered, the reduction of visibility during backward masking demonstrates that an internal representation that is responsible for the conscious awareness of the target does not arise instantaneously; in other words, the visual processing of the target takes time and can be disrupted by the mask before a conscious representation is finalized (e.g., Bachmann & Francis, 2014; Breitmeyer, 2007; Breitmeyer & Öğmen, 2006). Thus, investigating the mechanism of backward masking is directly linked to delineating how such a temporally evolving process underlies our conscious experience (see Aru & Bachmann, 2017; Bachmann, 1984).

In one kind of backward masking paradigm, referred to as metacontrast masking, a target (e.g., a disk) is surrounded by a mask (e.g., an annulus) that does not spatially overlap with the target but has inner contours that are adjacent to the contour of the target (e.g., Alpern, 1953; Breitmeyer & Öğmen, 2006). The effectiveness of metacontrast highly depends on the stimulus onset asynchrony (SOA) between the target and mask (Kahneman, 1967; but see Francis, 1997). Based on observations that metacontrast masking peaks at positive SOAs (when the target precedes the mask), several influential models have been proposed (Bachmann, 1984; Breitmeyer & Ganz, 1976; Breitmeyer & Öğmen, 2000).

### Common-onset masking and its mechanisms

However, another paradigm, common-onset masking, shows temporal properties that are quite different from those of metacontrast (Di Lollo, Enns, & Rensink, 2000; Enns & Di Lollo, 2000; for a review, see Goodhew, Pratt, Dux, & Ferber, 2013). While the SOA is fixed at zero across conditions, that is, the target and mask are always presented simultaneously, the mask offset is delayed relative to the target offset. Even though the target is easily seen when the target and mask are extinguished simultaneously, it becomes more difficult to see the target as the mask remains after the target offset. To avoid contour-based inhibitions that probably dominate metacontrast (Bridgeman, 1971; Francis, 1997), a sparse mask—such as four dots—remotely surrounding the target is mainly used in common-onset masking (e.g., Di Lollo et al., 2000; Filmer, Mattingley, & Dux, 2014; Llears & Moore, 2003; Pilling, Guest, & Andrews, 2019).

Di Lollo et al. (2000) proposed the object substitution theory as a mechanistic explanation, strongly relying on the idea that reentrant activities between higher and lower visual areas are essential to form durable conscious representations of visual stimuli (see Lamme, 2004; Lamme & Roelfsema, 2000). Object substitution is assumed to occur when higher-order representations are not significantly correlated with lower-order ones. In the case of common-onset masking, the initial feedforward sweep delivers information including both the target and mask to the higher areas; at the phase of reentrant activities, however, lower areas highly faithful to the inputs no longer represent the target but only the mask, so the mask-only representation is substituted for the older mask-plus-target representation.

In this theory, focusing attention on the target location plays a crucial role in shortening the duration of reentrant activities required for us to become initially aware of the target. In their computational model of object substitution (CMOS), however, Di Lollo et al. (2000) implemented the substitution process as a temporal integration of higher-order and lower-order representations, and attentional modulation as a temporal gate letting sensory information through (Reeves & Sperling, 1986; Sperling & Weichselgartner, 1995); thereby, their model should be regarded as a kind of attentional gating model (see Põder, 2013). The attentional involvement in common-onset masking assumed in this theory is consistent with findings indicating that masking becomes more effective as the set size (the total number of possible targets and distracters) increases and that precuing the location of the target releases it from masking (Di Lollo et al., 2000; Kotsoni, Csibra, Mareschal, & Johnson, 2007; Luiga & Bachmann, 2007; Neill, Hutchison, & Graves, 2002).

In contrast, Argyropoulos, Gellatly, Pilling, and Carter (2013) found little, if any, evidence of interaction between set size and mask duration when ceiling and floor performances were avoided, thereby suspecting that the apparent interaction between common-onset masking and attention would be an artifact of a ceiling effect that would occur at small set sizes (see also Filmer et al., 2014). Moreover, it was suggested that with the eccentricity of the target and distracters fixed across all set sizes, crowding between the target and distracters would exaggerate the masking effect only at sufficiently large set sizes, which could be another cause of apparent interaction (Camp, Pilling, Argyropoulos, & Gellatly, 2015). The computational model built by Põder (2013) also incorporates the independent contributions of attention and masking to the identification performance of the target. Not reentrant activities but two-stage noise addition processes are implemented in this model, and the sensitivity for the target is well predicted by a set-size-dependent signal-to-noise ratio at the preattentive stage and by a mask-duration-dependent signal-to-noise ratio at the attentive stage.

There is also another theory—object updating—that does not consider attentional involvement (e.g., Goodhew, 2017; Lleras & Moore, 2003; Pilling & Gellatly, 2010) but assumes that the contents of a single object-file are updated (see Kahneman, Treisman, & Gibbs, 1992). Accordingly, common-onset masking is effective when the target and mask are assigned to the same object-file because of the object correspondence between them. As some psychophysical evidence indicates, their correspondence can be established on the basis of apparent motion (Lleras & Moore, 2003; Pilling & Gellatly, 2010) and other grouping factors, such as common fate (Moore & Lleras, 2005). Conversely, cues facilitating object individuation between the target and mask, such as the difference in color (Moore & Lleras, 2005), the difference in luminance polarity (Luiga & Bachmann, 2008), and previewing the mask prior to the target onset (Neill et al., 2002), alleviate the masking effect.

Although these theories and models show some incompatibilities, they largely rely on some temporally evolving process prior to the final emergence of conscious perception—whether or not explicitly mentioned. The object substitution theory (Di Lollo et al., 2000), for example, explicitly expresses the temporal evolution of internal representation and its loss due to common-onset masking. Using the nomenclature of “global workspace” framework (e.g., Baars, 2005; Dehaene, Changeux, Naccache, Sackur, & Sergent, 2006), it can be stated that the internal representation of a masked target evolves from a “subliminal” form to a “preconscious” form, but not to a “conscious” form due to inattention (see Di Lollo et al., 2000; Põder, 2013). In other words, the target has sufficient potential to have access to the global workspace or to form a conscious representation as long as it is attended to. However, this kind of preconscious representation evolving over time remains a theoretical entity because the visibility reduction of a target only implies that the internal representation of the target is somehow weakened or lost, and it does not necessarily follow that the masked representation was in the middle of temporal evolution or was in a preconscious form. Therefore, to delineate the temporally evolving process behind the conscious awareness of objects, we must resort to a phenomenal attribute distinct from visibility.

### Temporal evolution of orientation appearance

We examined the appearance of a masked target. In our context, appearance denotes how the target looks like and is quantified as certain feature values, such as orientation, motion direction, depth, and color. An effective method to delineate the temporal evolution of appearance is to use a kind of illusion caused by contextual modulation. This is because if modulatory signals cause the internal representation of a certain feature value to evolve over time, then the effect of backward masking on the illusion can be psychophysically quantified as its reduction. The reduction of the illusion provides clear evidence that the masked representation was in the middle of temporal evolution if the feature value reported under backward masking is between what would be reported for the target alone and for the target within context but without a mask. Furthermore, the reduction of the illusion implies that the evolving representation can be preconscious because it is indeed consciously accessed and reported if and only if backward masking is applied.

Among various contextual illusions, we used orientation repulsion—a well-known phenomenon in which the orientation of a target is perceived as tilted away from that of a surrounding inducer (Clifford, 2014; Gibson, 1937; Schwartz, Hsu, & Dayan, 2007). Orientation repulsion strongly occurs when the orientation difference between the target and inducer is small (15°–30°), whereas larger differences (75°–80°) can sometimes induce an opposite effect to repulsion, called orientation assimilation (see Clifford, 2014; Westheimer, 1990). We investigated the common-onset masking effect on orientation repulsion in Experiment 1. To foreshadow, we found that masking did indeed reduce repulsion.

One of the physiological phenomena closely related to orientation repulsion is iso-orientation surround suppression. Specifically, responses of orientation-selective V1 neurons are suppressed when a stimulus with their preferred orientation is on their extraclassical receptive fields; however, this suppression occurs only when their classical receptive fields are also stimulated. Three mechanisms involving distinct levels of the processing hierarchy have been proposed for iso-orientation surround suppression (see Angelucci & Bressloff, 2006; Smith, 2006; Webb, Dhruv, Solomon, Tailby, & Lennie, 2005). The first is horizontal inhibition within a population of orientation-selective V1 neurons (e.g., Carpenter & Blakemore, 1973; DeAngelis, Freeman, & Ohzawa, 1994). Divisive normalization models, which successfully simulate both orientation repulsion and assimilation (Schwartz, Sejnowski, & Dayan, 2009; see also Goddard, Clifford, & Solomon, 2008), assume this kind of suppression. The second is feedback modulation from extrastriate cortices that have neurons with larger receptive fields than those of V1 neurons (Angelucci, Levitt, Walton, Hupé, Bullier, & Lund, 2002; Bair, Cavanaugh, & Movshon, 2003). The third is feedforward input from the lateral geniculate nucleus, in which iso-orientation surround suppression is observed (Ozeki, Sadakane, Akasaki, Naito, Shimegi, & Sato, 2004; Sillito, Cudeiro, & Murphy, 1993). Whereas the latter two (feedback and feedforward) mechanisms may be involved in a faster component of perceptual iso-orientation surround suppression, the first (horizontal) mechanism may be involved in a slower component (see Bair et al., 2003; Webb et al., 2005).

Considering these multitimescale components of perceptual iso-orientation surround suppression, we can argue about the time course of the contextual modulation underlying orientation repulsion. More specifically, the temporal evolution of the internal representation of the target orientation may be slow enough to be psychophysically traced. The psychophysical tracing of a temporally evolving representation is nontrivial because this is the only way to clarify whether the evolving representation is preconscious or subliminal, that is, whether it has potential to arise in one's awareness depending on the condition (for example, by paying attention; see Dehaene et al., 2006; Kanai, Walsh, & Tseng, 2010).

In Experiment 2, we orthogonally manipulated the mask offset time and the temporal mismatch between the target and inducer. Two previous studies manipulated the temporal mismatch in similar ways, aiming to reveal the temporal range of an inducer influencing the perceived orientation of a target. Durant and Clifford (2006) demonstrated that the strength of orientation repulsion peaked when the SOA between the target and inducer was zero and decreased with temporal mismatch. More specifically, repulsion was observed at least at all the examined SOAs from –200 to 50 ms. It was argued that repulsion occurred even when the asynchronous presentation of the inducer and target served as a cue facilitating the segregation between them. In Mareschal and Clifford's (2012) study, the inducer randomly switched its orientation in every frame (11.7 ms), and the target was added to only one frame in the middle of a trial sequence. Applying the reverse correlation method (see Ringach, 1998), the inducers, tilted 22.5° and 67.5° from the vertical presented within ±50 ms SOA, were found to yield repulsion and assimilation, respectively.

**Figure 1.**
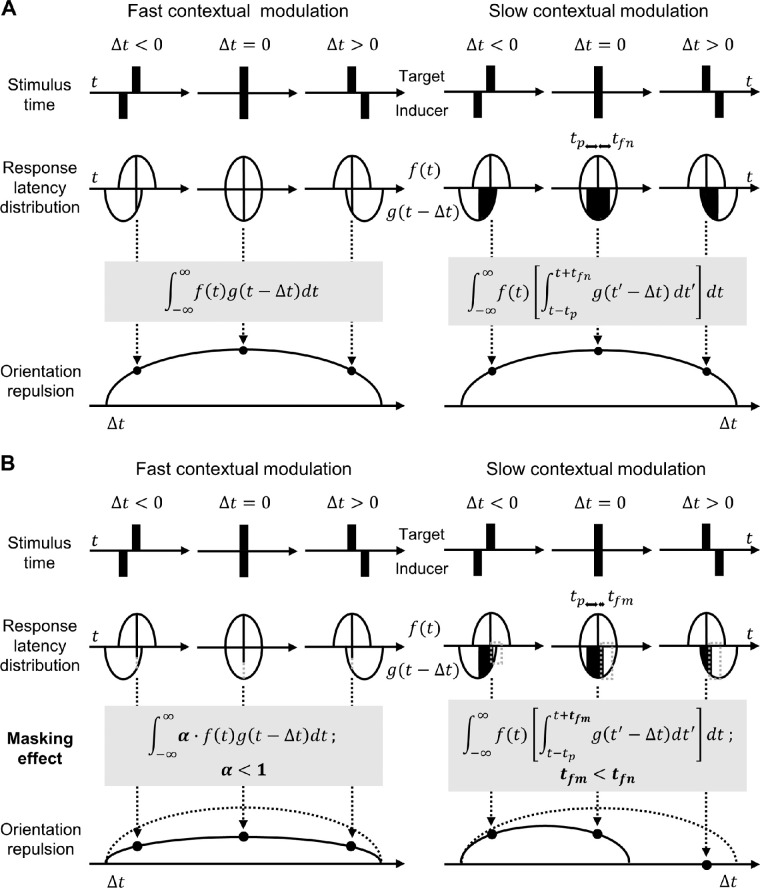
Two mechanisms underlying the temporal range of orientation repulsion. (A) Three types of temporal structures are shown: inducers are presented before (Δ*t* < 0), together with (Δ*t* = 0), and after (Δ*t* > 0) the target. The response latencies for the target and inducers are assumed to vary stochastically, and their distribution can be defined as *f*(*t*) for the target and *g*(*t* − Δ*t*) for the inducers. Left panel: If we assume that contextual modulation is fast, the strength of repulsion should be proportional to the probability that the responses to the target and inducers are simultaneous and thus follow the integral of the product of *f* and *g* (black lines perpendicular to the time axis) with respect to *t*. Right panel: If, on the other hand, we assume that contextual modulation is slow, the strength of repulsion should be proportional to the probability that the time of the response to the inducers is within some range around the time of the response to the target: the ranges toward the past and future relative to the time of the response to the target are denoted by *t_p_* and *t_fn_*, respectively, and the area of the black shade illustrates the integral within the square bracket. Thus, the strength of the repulsion should follow the integral of the product of *f* (black lines) and this area. In both cases, a qualitatively similar temporal function would be obtained (the row named “orientation repulsion”). (B) Backward-masking effect on temporal range of orientation repulsion. Left panel: If we assume that contextual modulation is fast, masking should disrupt contextual modulation regardless of Δ*t* (gray dotted lines) because the response to the inducers causing repulsion must be simultaneous with the response to the target. Here the masking effect is expressed as a multiplicative gain factor for repulsion (α < 1) for simplicity. Right panel: If, on the other hand, we assume that contextual modulation is slow, masking can disrupt the contribution of the response to the inducers at a specific time zone. Given that backward masking affects the visual processing of the target at a later time, the disruption can be localized to the later time zone (gray dotted zones). This disruption is expressed as the reduction of *t_fn_* to *t_fm_*. Consequently, repulsion is reduced more when Δ*t* ≥ 0 than when Δ*t* < 0. Therefore, qualitatively different temporal functions would be obtained, depending on the assumption of the time course of contextual modulation.

However, both fast and slow contextual modulation mechanisms can account for the aforementioned temporal range of repulsion by introducing the stochastic variability of differential response latency (Figure 1A), which has been proposed as a parsimonious explanation for the flash-lag effect (Murakami, 2001a, 2001b; Whitney & Murakami, 1998). The flash-lag effect refers to the perceived temporal lag of a briefly flashed stimulus behind a moving stimulus, and the estimated lag has a distribution of 50–100 ms, which is tentatively explained by the trial-to-trial variability of the differential response latency between the flash and moving stimuli (Murakami, 2001a, 2001b). Similarly, in the situation of orientation repulsion, the differential response latency between the target and inducer might vary stochastically around 0 ms because they are both static. Even if the inducer is asynchronous with the target, repulsion would occur if the neural response to the inducer is simultaneous with the response to the target. In this way, fast modulation can yield repulsion that apparently extends over time (Durant & Clifford, 2006; Mareschal & Clifford, 2012). On the other hand, slow modulation can yield repulsion not only when the neural responses to the target and inducer occur simultaneously but also when they do asynchronously. Consequently, repulsion as a function of time is expected to follow the same pattern as when fast modulation is assumed (for more details, see Figure 1A).

Nevertheless, if backward masking operates on the target, fast and slow contextual modulation mechanisms produce different psychophysical predictions (Figure 1B). If the modulation is fast, and thus the temporal range of repulsion is fully explained by the stochastic variability of the differential response latency between the target and inducer, masking should disrupt modulation regardless of when the inducer is presented relative to the target because modulation occurs only when the response to the inducer is simultaneous with that to the target. In contrast, if the modulation is slow, the masking effect on contextual modulation can be temporally localized because modulation occurs when the response to the inducer is within a certain range around the time of that to the target. Given that backward masking affects the visual processing of the target at a later time, contextual modulation from an inducer presented not before but after the target onset time could be selectively disrupted (for more details, see Figure 1B). Experiment 2 was conducted to see which idea was the case.

In addition to repulsion, we quantified orientation discriminability in both experiments to determine which of the three alternative scenarios was the case. First, discriminability might be reduced by backward masking, as has been demonstrated in previous studies (e.g., Agaoglu, Agaoglu, Breitmeyer, & Öğmen, 2015; Agaoglu, Breitmeyer, & Öğmen, 2016; Harrison, Rajsic, & Wilson, 2016). Second, discriminability might be immune to masking, as has been reported in previous studies showing parameter dependences of performance during masking (see e.g., Bouvier & Treisman, 2010; Goodhew, Edwards, Boal, & Bell, 2015), since our choice of stimulus parameters deliberately left the target detectable so as not to hinder orientation judgment per se. Third, discriminability might be improved along with a reduction of repulsion due to masking because orientation repulsion could introduce a loss of orientation discriminability (see Solomon & Morgan, 2009).

## Experiment 1

### Methods

Eleven observers (eight males and three females; age: 19–24 years) with normal or corrected-to-normal vision participated. All but one, the first author, were unaware of the purpose of the experiment. One naïve observer exhibiting the amount of illusion deviating from the interobserver mean by more than three SDs was excluded as an outlier. The study adhered to the Declaration of Helsinki and was approved by the ethics committee of the Graduate School of Humanities and Sociology at the University of Tokyo. Prior to the experiment, all the observers provided written informed consent.

The experimental programs written in MATLAB R2018A (Mathworks, Natick, MA, USA) with Psychophysics Toolbox version 3.0.14 (Brainard, 1997; Kleiner, Brainard, & Pelli, 2007; Pelli, 1997) were executed on a computer (Apple MacPro Late 2013 equipped with an AMD FirePro D700 graphic card). All stimuli were displayed on a uniform gray background (20.8 cd/m^2^) of a CRT monitor (Mitsubishi Electric RDF223H) with a spatial resolution of 800 × 600 pixels and a refresh rate of 60 Hz. The monitor was gamma-corrected using a photometer (Cambridge Research Systems ColorCAL, Rochester, UK). The observers fixed their heads on a chin-and-head rest 57 cm away from the monitor with both eyes opened during the experiment.

A 1.2 dva × 1.2 dva (degrees of visual angle) white cross was used as a fixation point. The target and inducers for repulsion were Gabor patches that were identical except for their orientation. Their spatial frequency was 2 cpd, and the SD of the Gaussian window was 0.42 dva. They were presented with 88% contrast. The target orientation was chosen in a random order from –8°, –6°, –4°, –2°, 0°, 2°, 4°, 6°, or 8°, where the positive indicates a clockwise tilt from the vertical (i.e., 0°). In contrast, each of the eight Gabor patches composing the inducers was tilted 20° in the clockwise (CW) condition and –20° in the counterclockwise (CCW) condition to induce orientation repulsion to the target. These eight patches were evenly spaced along a virtual circle surrounding the target patch. In the baseline condition, 20° and –20° patches were arranged in an interleaved manner to cancel out the repulsion from each other. The target patch was presented at 7.5 dva to the left or right of the fixation cross to prevent observers from prefocusing attention on the target location, as paying attention could lessen the masking effect (see Di Lollo et al., 2000; Neill et al., 2002). On the other hand, the circularly arranged inducers were presented on both sides, with each side centered at the target location, and therefore uninformative about the side at which the impending target would appear. The center-to-center distance between the target and each Gabor patch composing the inducers was 4.0 dva. As a mask, we used four dots (0.35 dva × 0.35 dva square each) located at each vertex of a virtual upright square (2.8 dva × 2.8 dva) concentric to the target; thus, the dots were located between the target and inducers. The center-to-center distance between the target and each dot was 2.0 dva.

**Figure 2.**
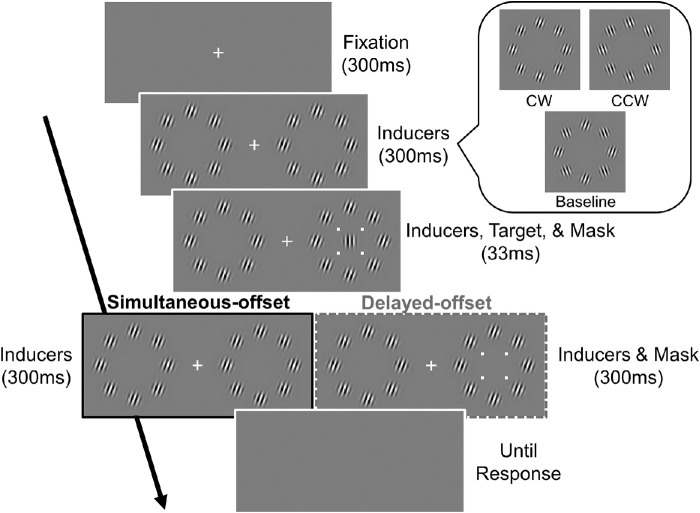
Time course of each trial in Experiment 1. In the simultaneous-offset condition, the target Gabor patch and four-dot mask disappeared together (left). In the delayed-offset condition, the mask disappeared 300 ms after the target offset (right). The three types of inducers (for the CW, CCW, and baseline conditions) are shown in the inset.

At the beginning of each trial, the fixation cross was presented alone for 300 ms, after which inducers were added (Figure 2). The target and four-dot mask were added 300 ms later. The target duration was 33 ms. In the simultaneous-offset condition, the mask disappeared together with the target, while in the delayed-offset condition, it remained for 333 ms. All the stimuli disappeared 300 ms after the target disappeared, and the observers responded by pressing a key at their own pace to indicate whether the target was tilted clockwise or counterclockwise from the vertical. As soon as the response was recorded, a beep sound signaled the next trial that would start 2000 ms later. No feedback was provided.

In one experimental session, 108 trials for all combinations of the conditions, namely, three inducer orientations (CW, CCW, and baseline) × two mask types (simultaneous and delayed) × nine target orientations (–8° to 8°) × two target locations (left and right), were performed in a random order. Each observer undertook 24 such sessions; thus, the total number of trials per observer was 2592. There was an adequate rest interval between consecutive sessions.

### Results and discussion

We merged the trials for the two target locations because they yielded no essential differences, and out of 48 such repetitive responses, we calculated the proportion of trials in which the target was perceived as tilted clockwise and plotted it as a function of the physical orientation of the target. The maximum likelihood fitting to a logistic function (Equation 1) was performed on the data using the Palamedes Toolbox (Kingdom & Prins, 2016).

(1) $$Logistic\left(x|\alpha ,\beta ,\lambda \right)=\frac{\lambda }{2}+\frac{1-\lambda }{1+\exp[-\beta (x-\alpha )]}$$

where α, β, and λ denote the point of subjective verticality (PSV), slope, and lapse rate, respectively. For each observer, psychometric functions were derived for the six conditions: three inducer orientations (CW, CCW, and baseline) × two mask types (simultaneous and delayed). While PSVs were allowed to vary across the six conditions, slope and lapse rate were constrained across the CW, CCW, and baseline conditions to avoid overfitting, but they were allowed to vary between the simultaneous-offset and delayed-offset conditions because these parameters could be affected by masking (see Agaoglu et al., 2015; Agaoglu et al., 2016; Harrison et al., 2016).

**Figure 3.**
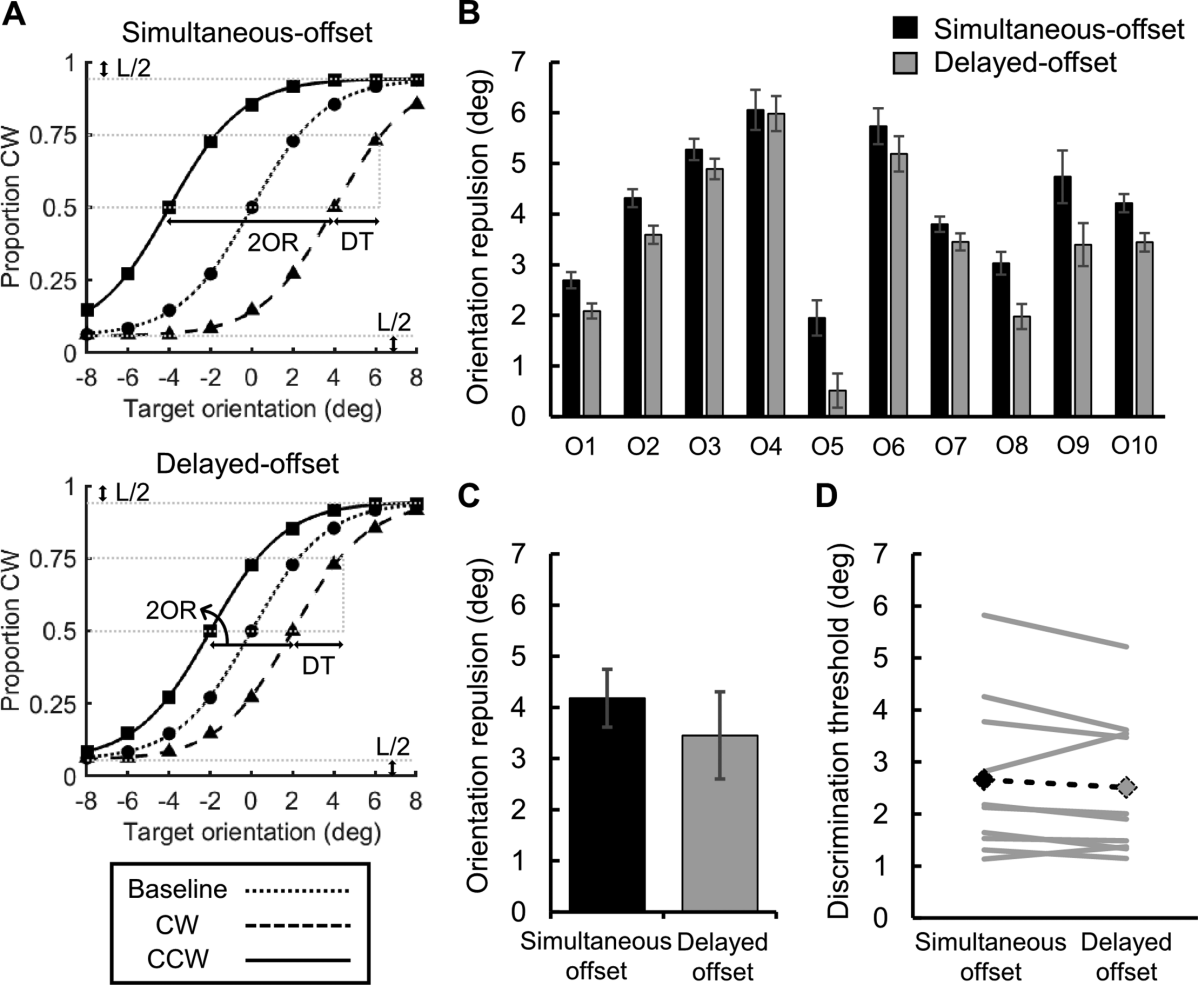
Results of Experiment 1. (A) Psychometric functions fitted against virtual data for the simultaneous-offset (above) and delayed-offset (below) conditions. OR, DT, and L indicate the estimated orientation repulsion, discrimination threshold, and lapse rate, respectively. (B) Orientation repulsion for each of 10 observers (O1 to O10). The error bars indicate the standard errors estimated by bootstrap simulations. (C) Interobserver mean of orientation repulsion. The black and gray bars indicate the simultaneous-offset and delayed-offset conditions, respectively. The error bars indicate the standard error of the means. (D) Discrimination threshold for orientation. The solid gray lines indicate individual data, and the dashed black line indicates the interobserver mean.

Orientation repulsion should lead to a rightward shift of the CW curve and a leftward shift of the CCW curve with respect to the baseline curve (Figure 3A). The strength of repulsion was determined as half the distance between the PSVs for the CW and CCW conditions, and the discrimination threshold, an index of discriminability, was determined as the difference between the physical target orientations corresponding to the proportions of 75% and 50% (i.e., PSV). A paired *t*-test revealed that orientation repulsion in the delayed-offset condition (*M* = 3.45, *SD* = 1.65) was significantly weaker than that in the simultaneous-offset condition (*M* = 4.16, *SD* = 1.38; *t*(9) = 5.52, *p* < .001, *d_z_* = 1.75), but significantly stronger than zero (*t*(9) = 6.60, *p* < 0.001, *d_z_* = 2.09). These indicate that the delay of the mask offset made the orientation appearance of the target more faithful to the light distribution on the retina (see Figures 3B, C).

In contrast, the discrimination thresholds did not significantly differ regardless of mask type, as shown in Figure 3D (delayed-offset: *M* = 2.51, *SD* = 1.37; simultaneous-offset: *M* = 2.66, *SD* = 1.52; *t*(9) = 1.19, *p* = 0.263, *d_z_* = 0.38). We detected no significant difference in the discrimination thresholds, even when they were newly estimated only from the data in the baseline condition, in which no repulsion could occur in principle (delayed-offset: *M* = 2.63, *SD* = 1.49; simultaneous-offset: *M* = 2.77, *SD* = 1.78; *t*(9) = 0.43, *p* = 0.679, *d_z_* = 0.14). These results indicate that the delay of the mask offset does not reduce discriminability in our situation. Furthermore, we confirmed that lapse rates did not differ between the simultaneous-offset and delayed-offset conditions (*p* = 0.195; Wilcoxon signed-rank test).

In Experiment 1, in accordance with most previous studies on common-onset masking, a four-dot mask composing a virtual upright square was used. Although this kind of mask was optimal for minimizing the contour-based interaction with the target (e.g., Francis, 1997), our particular experimental situation might be susceptible to the potential confound that the physical arrangement of the four-dot mask modifies the target's apparent orientation. As the virtual upright square subjectively had vertical components, it might serve as a cardinal reference and compromise orientation repulsion. If this were the case, the reduction of repulsion might have occurred simply because the mask as a reference remained longer after the target disappeared. To address this issue, we conducted two control experiments (see Supplementary Materials). In Control Experiment A, the virtual square composed of the four-dot mask was tilted randomly and could not be used as a vertical reference (Figure S1A). In Control Experiment B, two black vertical bars were physically added next to the target location on each side. The bars appeared and disappeared together with the inducers regardless of mask type; thus, the available time of this vertical reference was equivalent between conditions (Figure S1B). Even in these control experiments, we found that repulsion in the delayed-offset condition was significantly weaker than that in the simultaneous-offset condition (Experiment A: *t*(8) = 2.37, *p* = 0.045, *d_z_* = 0.79, Figure S2A; Experiment B: *t*(9) = 3.40, *p* = 0.008, *d_z_* = 1.08, Figure S2B), but significantly stronger than zero (Experiment A: *t*(8) = 7.73, *p* < 0.001, *d_z_* = 2.58; Experiment B: *t*(9) = 8.41, *p* < 0.001, *d_z_* = 2.66; one-sample *t*-tests). Therefore, the physical arrangement of the four-dot mask does not fully explain our findings; rather, some mechanisms related to masking contribute to the reduction of repulsion. In addition, as in Experiment 1, there was no significant difference in the discrimination threshold (Experiment A: *t(8)* = 0.27, *p =* 0.793, *d_z_* = 0.09, Figure S2C; Experiment B: *t*(9) = 1.93, *p* = 0.085, *d_z_* = 0.61, Figure S2D) or lapse rate (Experiment A: *p* = 0.375; Experiment B: *p* = 0.250; Wilcoxon signed-rank tests).

## Experiment 2

In Experiment 1, we found that common-onset masking reduced orientation repulsion. However, the interaction between masking and the temporally evolving representation of the target orientation underlying this reduction remains to be clarified. In Experiment 2, we manipulated the time zone during which the inducers were presented and examined how much common-onset masking affected contextual modulation from the inducers at each time zone. Repulsion will be reduced at all the time zones if the modulation is fast, while it will be selectively reduced only at later time zones if the modulation is slow (see Figure 1).

### Methods

Sixteen observers (10 males and six females; age: 20–45 years) with normal or corrected-to-normal vision participated. All but two, including the first author, were unaware of the purpose of the experiment. The ethical procedure was identical to that of Experiment 1.

The apparatus was the same as that used in Experiment 1. As for the stimuli, the Gabor parameters except for orientation were equivalent to those in Experiment 1. We reduced the number of target orientations to four (–3°, –1°, 1°, and 3°) because the number of trials became excessive due to the multiple presentation timings of inducers, as explained below. Furthermore, the orientation repulsion we demonstrated above was so robust that we were able to omit the baseline condition here; thus, we only included the CW and CCW conditions in which all inducers were tilted 20° and –20°, respectively, from the vertical. To avoid possible confounds of the virtual verticality of the mask, the mask configuration subtly differed from that in Experiment 1. More specifically, we used five dots (0.35 dva × 0.35 dva square each) located at the vertices of a virtual inverted equilateral pentagon, which had no vertical component and was concentric to the target. The center-to-center distance between the target and each dot was 2.0 dva, exactly the same as in Experiment 1.

**Figure 4.**
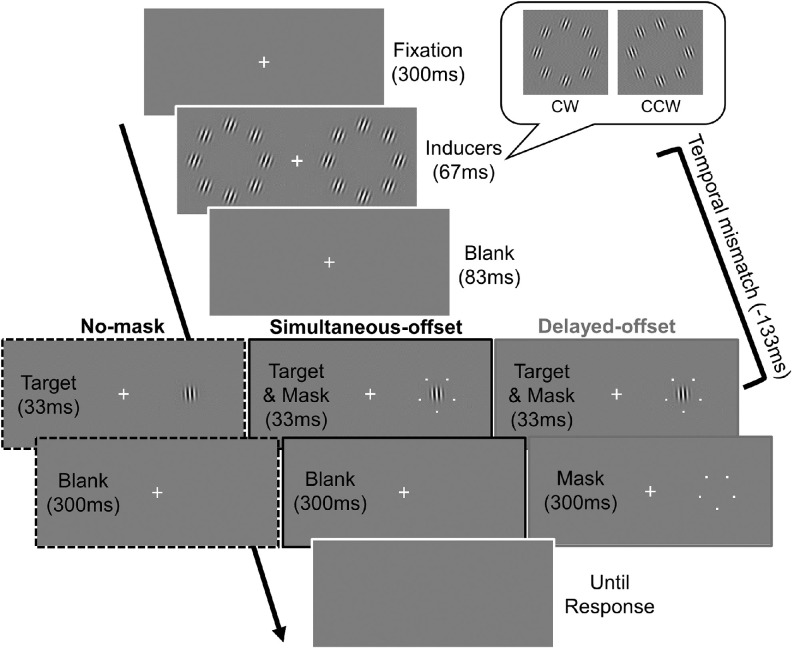
Time course of each trial in Experiment 2. The case in which the temporal mismatch between the target and inducers was –133 ms is shown. In the no-mask condition, the five-dot mask never appeared (left). In the simultaneous-offset condition, the target Gabor patch and mask disappeared together (center). In the delayed-offset condition, the mask disappeared 300 ms after the target offset (right). Two types of inducers (for the CW and CCW conditions) are shown in the inset.

In each trial, the fixation cross was presented for 783 ms, and the target appeared 450 ms after the onset of the fixation cross, irrespective of the time of the inducers or the mask duration (Figure 4). Most importantly, the inducers were only briefly presented (66 ms long) around the time of the target (33 ms long) to clarify the temporal property of the reducing effect of common-onset masking on orientation repulsion. There were five levels (–133, –67, 0, 67, and 133 ms) of temporal mismatch, which was defined as the difference between the midpoints of the durations of the target and inducers: the positive indicates that the target was followed by the inducers.

We added a no-mask condition in which the five-dot mask never appeared. This was motivated by previous studies showing that a four-dot mask could lower target discriminability even when their onset and offset were in common (Enns & Di Lollo, 1997; Nakamura, Lavrenteva, & Murakami, 2020). If this is the case in our stimulus configuration, the presence of the mask may affect the discriminability and/or appearance of target orientation, independent of common-onset masking.

In every two experimental sessions, 240 trials for all combinations of the conditions, namely, two inducer orientations (CW and CCW) × three mask types (simultaneous-offset-mask, delayed-offset-mask, and no-mask) × five temporal mismatches (–133, –67, 0, 67, and 133 ms) × four target orientations (–3°, –1°, 1°, and 3°) × two target locations (left and right), were performed in a random order. Each observer undertook 20 such sessions; thus, the total number of trials per observer was 2400. There was an adequate rest interval between consecutive sessions.

### Results and discussion

**Figure 5.**
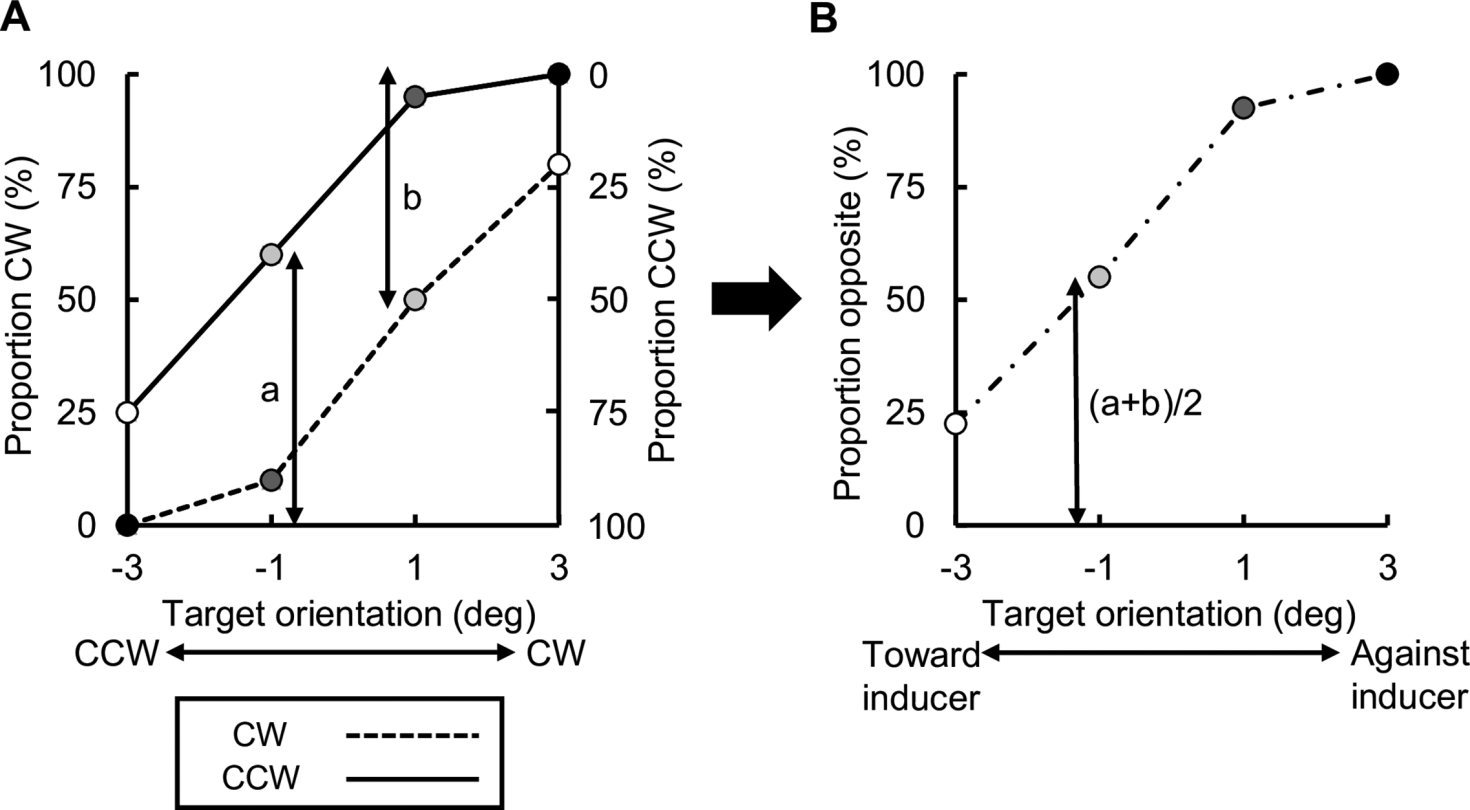
Derivation of proportion opposite. (A) Proportion of trials in which the target was perceived as tilted clockwise plotted as a function of target orientation (one naïve observer's data at –133 ms in the simultaneous-offset condition). The dashed and solid curves indicate the conditions in which the inducers were tilted CW and CCW, respectively. The proportions of trials in which the target was perceived as tilted against inducer orientation (“proportion opposite”) were calculated by flipping and merging the data of the CW condition to those of the CCW condition. (B) Proportion opposite as a function of target orientation. The gray levels of the symbols in B correspond to those in A. For example, the proportion opposite at –1°, the length of the arrow in B is the average of the lengths of the two arrows in A, that is, “a” (Proportion CW in the CCW condition) and “b” (Proportion CCW in the CW condition).

**Figure 6.**
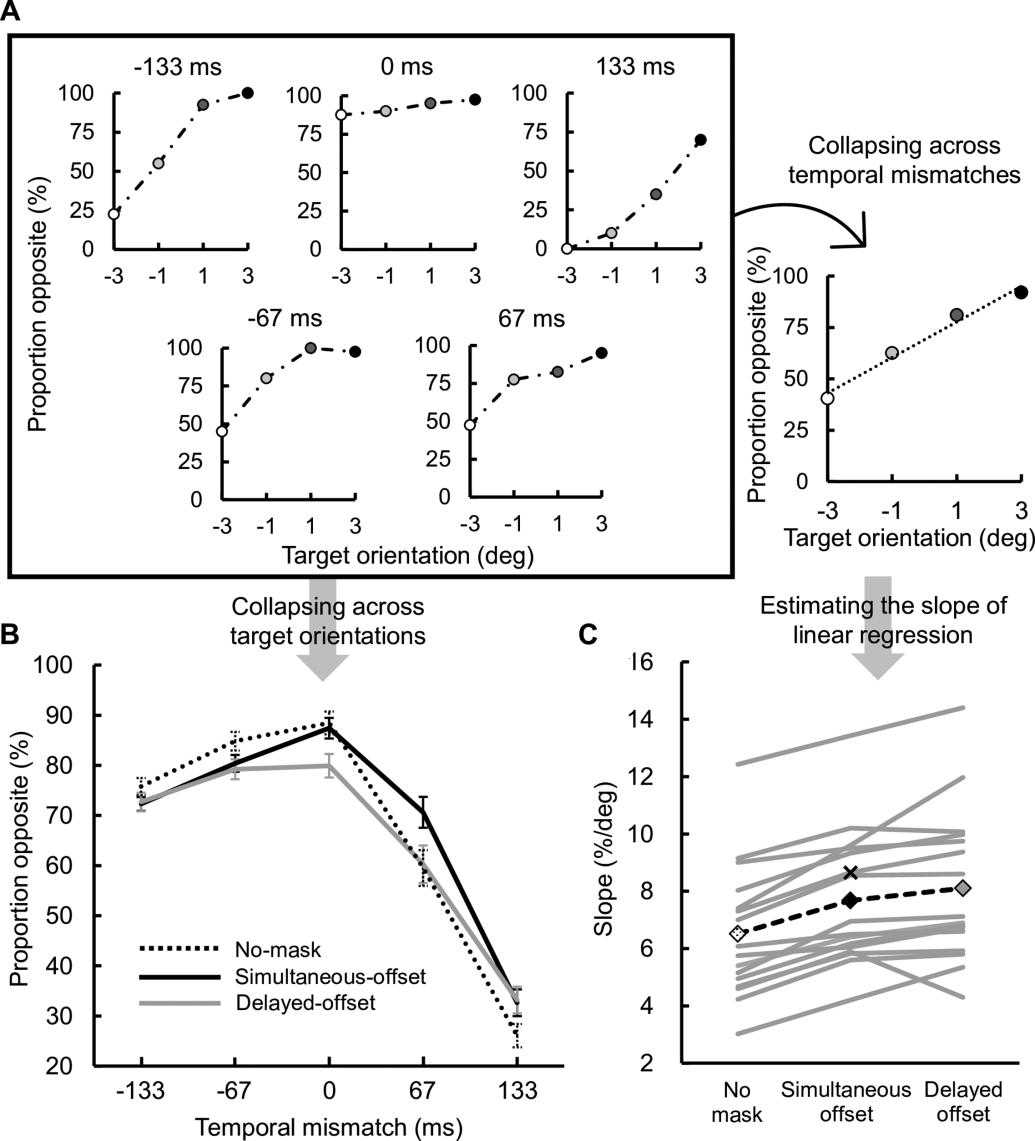
Results of Experiment 2. (A, left) Proportion opposite as a function of target orientation at each temporal mismatch. The data in the simultaneous-offset condition for the naïve observer whose data also appear in Figure 5 are shown. (A, right) The proportion opposite data shown on the left side were collapsed across temporal mismatches and were fitted with a linear function. (B) The proportion opposite data shown above were collapsed across target orientations and averaged across observers. The black dashed, black solid, and gray solid curves indicate the no-mask, simultaneous-offset, and delayed-offset conditions, respectively. The error bars indicate the standard error of the means. (C) Orientation discriminability was estimated as the slope of the linear regression. The solid curves indicate the slope of each observer, whose value denotes the change in proportion opposite per degree of target orientation. The dashed black curve indicates the interobserver mean. The cross symbol indicates the slope for the example shown in A.

First, as in Figure 3 for Experiment 1, we plotted the proportion of trials in which the target was perceived as tilted clockwise as a function of target orientation (Figure 5A). According to Figure 3, orientation repulsion is equivalent to a rightward shift of the CW curve relative to the CCW curve. Next, from these data for the CW and CCW conditions, we averaged the proportion of trials in which the target was perceived as tilted against the inducers (hence named “proportion opposite”). Specifically, the proportion CCW in the CW condition (b in Figure 5A) was flipped and merged to the proportion CW in the CCW condition (a) to obtain the data of proportion opposite at each target orientation (Figure 5B); positive orientations now denote that the target was tilted away from the inducers. Thus, instead of estimating a PSV, which would have required more than four data points to avoid overfitting, we assessed the strength of orientation repulsion in terms of the proportion opposite collapsed across the target orientation at each temporal mismatch (Figures 6A and B). The collapsed proportion opposite is qualitatively similar to orientation repulsion in Experiment 1 in that both reflect how far the CW and CCW curves are away from each other. The results of one-sample *t*-tests showed that the proportion opposite data at all the mismatches were significantly higher than 50%, except at 133 ms mismatch, at which they were significantly lower than 50% (*p*s < 0.05; adjusted by Holm's method), indicating that orientation repulsion occurred at –133, –67, 0, and 67 ms; however, the observers’ responses were biased toward the inducer orientation at 133 ms. Since no qualitative difference was detected in the results, whether the following analyses be performed on the linear proportions or their log-odds, only the former results are reported hereafter.

We performed a two-way repeated measures ANOVA with mask type and temporal mismatch as factors. Because the data did not pass the multisample sphericity test (Mendoza, 1980), the degree of freedom was adjusted using Greenhouse-Geisser's ε. The main effects of mask type and temporal mismatch and their interaction were all significant (*F*(1.4, 21.1) = 9.79, *p* = 0.002, $\eta_{p}^{2}$ = 0.395; *F*(1.9, 28.5) = 136.39, *p* < 0.001, $\eta_{p}^{2}$ = 0.901; *F*(4.1, 62.0) = 16.13, *p* < 0.001, $\eta_{p}^{2}$ = 0.518). A trend analysis with orthogonal polynomials revealed that the main effect of temporal mismatch displayed a significant quadratic trend (*F*(1, 15) = 296.96, *p* < 0.001, $\eta_{p}^{2}$ = 0.952).

The simple effects of mask type were also significant at all temporal mismatches (–133 ms: *F*(1.6, 23.6) = 4.73, *p* = 0.003, $\eta_{p}^{2}$ = 0.240; –67 ms: *F*(1.7, 25.2) = 14.05, *p* < 0.001, $\eta_{p}^{2}$ = 0.484; 0 ms: *F*(1.5, 23.0) = 17.56, *p* < 0.001, $\eta_{p}^{2}$ = 0.539; 67 ms: *F*(1.7, 26.0) = 15.19, *p* < 0.001, $\eta_{p}^{2}$ = 0.503; 133 ms: *F*(2.0, 29.6) = 16.18, *p* < 0.001, $\eta_{p}^{2}$ = 0.519); thus, multiple comparisons (Holm, 1979) were performed across the three mask types at each temporal mismatch. At –133 ms, the proportion opposite in the simultaneous-offset condition was significantly smaller than that in the no-mask condition (*t*(15) = 3.58, *p* = 0.008, *d_z_* = 0.89), but they did not differ from that in the delayed-offset condition (*t*(15) = 0.33, *p* = 0.743, *d_z_* = 0.08; *t*(15) = 2.04, *p* = 0.119, *d_z_* = 0.51). At –67 ms, the proportion opposite data in the simultaneous-offset and delayed-offset conditions did not differ (*t*(15) = 1.30, *p* = 0.213, *d_z_* = 0.32), but they were significantly smaller than that in the no-mask condition (*t*(15) = 4.08, *p* = 0.002, *d_z_* = 1.02; *t*(15) = 4.24, *p* = 0.002, *d_z_* = 1.06). At 0 ms, the proportion opposite data in the simultaneous-offset and no-mask conditions did not differ (*t*(15) = 0.99, *p* = 0.340, *d_z_* = 0.25), but they were significantly greater than that in the delayed-offset condition (*t*(15) = 4.41, *p* = 0.001, *d_z_* = 1.10; *t*(15) = 4.64, *p* = 0.001, *d_z_* = 1.16). At 67 ms, the proportion opposite data in the delayed-offset and no-mask conditions did not differ (*t*(15) = 0.31, *p* = 0.764, *d_z_* = 0.08), but they were significantly smaller than that in the simultaneous-offset condition (*t*(15) = 4.16, *p* = 0.002, *d_z_* = 1.04; *t*(15) = 6.32, *p* < 0.001, *d_z_* = 1.58). At 133 ms, biases opposite to repulsion were observed in all conditions, as mentioned above. The biases in the simultaneous-offset and delayed-offset conditions did not differ (*t*(15) = 0.38, *p* = 0.709, *d_z_* = 0.09), but they were significantly smaller than that in the no-mask condition (*t*(15) = 4.62, *p* < 0.001, *d_z_* = 1.16; *t*(15) = 5.43, *p* < 0.001, *d_z_* = 1.36).

The simple effects of temporal mismatch were also significant for all mask types (simultaneous-offset: *F*(2.3, 34.7) = 121.82, *p* < 0.001, $\eta_{p}^{2}$ = 0.890; delayed-offset: *F*(1.8, 27.4) = 74.21, *p* < 0.001, $\eta_{p}^{2}$ = 0.832; no-mask: *F*(2.1, 30.1) = 162.56, *p* < 0.001, $\eta_{p}^{2}$ = 0.916). The proportion opposite was greatest at 0 ms and decreased as the target and inducers became asynchronous in all conditions, as confirmed by multiple comparisons (Holm, 1979). As for comparisons between adjacent temporal mismatches, all pairs showed a significant difference (*p*s < 0.01), except that in the delayed-offset condition, the data did not differ between –67 and 0 ms (*p* = 0.724). Correspondingly, in each condition, the proportion opposite displayed significant quadratic (i.e., inverted U-shaped) trends against the temporal mismatch (simultaneous-offset: *F*(1, 15) = 281.28, *p* < 0.001, $\eta_{p}^{2}$ = 0.949; delayed-offset: *F*(1, 15) = 122.73, *p* < 0.001, $\eta_{p}^{2}$ = 0.891; no-mask: *F*(1, 15) = 300.01, *p* < 0.001, $\eta_{p}^{2}$ = 0.952).

Next, the proportion opposite data were collapsed across the temporal mismatches to estimate discriminability, which we naturally assumed to be immune to the time when the inducers were presented. The discriminability for each mask type was determined as the slope of the linear regression for proportion opposite as a function of target orientation (Figure 6C). A one-way repeated measures ANOVA with mask type as a factor was performed on the discriminability data. Again, the degree of freedom was adjusted using Greenhouse-Geisser's ε because of the violation of multisample sphericity. The main effect of mask type was significant (*F*(1.28, 19.13) = 32.90, *p* < 0.001, $\eta_{p}^{2}$ = 0.687). Multiple comparisons (Holm, 1979) confirmed that discriminability was significantly lower (i.e., slope was closer to zero) in the no-mask condition than in the simultaneous-offset and delayed-offset conditions (*t*(15) = 9.61, *p* < 0.001, *d_z_* = 2.40; *t*(15) = 6.08, *p* < 0.001, *d_z_* = 1.52); however, the difference between the latter two conditions did not reach significance (*t*(15) = 2.11, *p* = 0.052, *d_z_* = 0.53). Therefore, as in Experiment 1, common-onset masking did not hamper discriminability; the presence of the five-dot mask rather improved it in Experiment 2. The same results were obtained even when log-transformed slopes were tested (main effect: *F*(1.47, 22.03) = 29.72, *p* < 0.001, $\eta_{p}^{2}$ = 0.665; pair-wise comparisons: *t*(15) = 8.02, *p* < 0.001, *d_z_* = 2.00; *t*(15) = 5.85, *p* < 0.001, *d_z_* = 1.46; *t*(15) = 1.57, *p* = 0.138, *d_z_* = 0.39).

The results of Experiment 2 are summarized in five points as follows. First, although orientation repulsion occurred whether the inducers and target were simultaneous or not, the strength of the illusion decreased as the temporal mismatch increased. The inverted U-shaped temporal function of repulsion is qualitatively similar to that depicted by Durant and Clifford (2006) in terms of width and asymmetry. In contrast, Mareschal and Clifford (2012) examined the repulsion with a higher temporal resolution and obtained a narrower and more symmetric temporal function. This shape difference in the temporal functions for repulsion may be derived from the visible persistence of the inducers. The inducer used in Mareschal and Clifford's (2012) study randomly switched its orientation every 12 ms; thus, its visible persistence was extinguished at every switch, but the inducers used in Durant and Clifford's (2006) and our studies had a single orientation per trial and were never masked. Given that visible persistence prolonged the perceived duration of the inducers, our broader and more negatively skewed temporal functions than that in Mareschal and Clifford's (2012) study may be accounted for by such an effective duration.

Second, and most importantly, the difference in the proportion opposite between the simultaneous-offset and delayed-offset conditions was localized at 0 and 67 ms, demonstrating that contextual modulation from later inducers was selectively interrupted by common-onset masking. This finding cannot be explained simply by the stochastic variability of the differential response latency for the target and inducers. We would argue the involvement of some temporally evolving slow process, but the detailed mechanism we propose will be discussed in the General discussion section.

Third, the difference in the proportion opposite between the no-mask and simultaneous-offset conditions was localized at –133 and –67 ms, indicating that the presence of the mask selectively interfered with contextual modulation from earlier inducers. This may be consistent with previous studies demonstrating that a transient mask can interrupt the filling-in process (Motoyoshi, 1999; Paradiso & Nakayama, 1991). Motoyoshi (1999) found that when an annulus mask known to exert metacontrast masking was superimposed on a texture pattern consisting of iso-oriented line segments, the texture inside the mask contour became less visible, suggesting that the transient mask could block the sluggish orientation filling-in process. A similar phenomenon was observed during brightness filling-in; especially in the brightness dimension, even a mask with minimal contours (similar to the five dots in our experiment) could block the filling-in process (Paradiso & Nakayama, 1991). It is possible that the process of contextual modulation evolving as sluggishly as the orientation filling-in was interrupted by the transient signals caused by the mask onset.

Fourth, we detected no decrease in discriminability due to common-onset masking, as in Experiment 1; however, we found that it rather improved in the presence of the mask. In our situation, the onset of the five dots might also have served as a location cue to the target. In any case, this improvement should be independent of the common-onset masking effect because discriminability was improved in both simultaneous-offset and delayed-offset conditions to the same extent.

Fifth, at 133 ms, a bias opposite to repulsion was seen; in other words, the observers’ responses were biased toward the inducer orientation. A similar tendency was also found by Durant and Clifford (2006). At this temporal mismatch, the target orientation might have been attracted to the inducer orientation because of the establishment of object correspondence between the target and inducers (Kawabe, 2012). Alternatively, observers might have merely taken a response strategy of reporting the inducer orientation when they found it hard to indicate the target orientation beyond guessing. The reason why the bias was stronger in the no-mask condition than in the simultaneous-offset and delayed-offset conditions may be that this strategy was taken more frequently because of lower discriminability in the no-mask condition than in the other conditions, as mentioned above. In a similar vein, it might have also been the case at 67 ms that lower discriminability caused a stronger bias toward the inducer orientation in the no-mask condition, making its proportion opposite apparently smaller than that in the simultaneous-offset condition.

## General discussion

### Summary of results

In the present study, we investigated how common-onset masking influences orientation discriminability and appearance. In Experiment 1, we found that common-onset masking reduced orientation repulsion. In Experiment 2, we found that this reduction was mainly caused by inducers presented simultaneously with or later than the target. In contrast to appearance, the orientation discriminability was never reduced by masking. Using backward masking enabled us to psychophysically trace the temporally evolving representation underlying orientation repulsion. Moreover, our findings provide direct evidence of preconscious representations in the middle of evolution that can be consciously accessed if and only if masking operates.

### Possible reasons why orientation discriminability was not lowered by masking

Backward masking can reduce the discriminability of the target. (Bachmann & Francis, 2014; Breitmeyer & Öğmen, 2006). However, through the two experiments, we consistently found no evidence of reduced discriminability due to common-onset masking. One possibility is that in the delayed-offset condition, masking reduced discriminability while the reduction of repulsion improved it (see Solomon & Morgan, 2009), and, because these effects cancelled each other, discriminability was apparently the same as in the simultaneous-offset condition. However, this less parsimonious explanation is not viable because in Experiment 1, discriminability did not differ even when it was estimated only from the baseline data, from which we concluded that masking did not reduce discriminability even when no repulsion occurred. Here we consider why discriminability was not reduced by masking, aside from repulsion. This can be expected to some extent because our stimulus parameters were deliberately adjusted prior to the experiments so that the target could be at least detected even during masking, that is, in the delayed-offset condition. This prior adjustment for the feasibility of the discrimination task may have resulted in comparable performances in both simultaneous-offset and delayed-offset conditions.

Other reasons why discriminability was not reduced in our situation can be inferred from previous findings. Bouvier and Treisman (2010) examined the effect of common-onset masking on task performance in judging the feature (color or orientation) of a target. In their search display, multiple crosses, each of which was composed of one white bar and one nonwhite bar, were presented. The target was designated as a nonwhite bar surrounded by a four-dot mask; in other words, it was defined by its location and color rather than by its orientation. Thus, orientation judgments required feature binding, while color judgments did not. Masking lowered performance in orientation judgment, but not in color judgment. On the other hand, if the target was followed by a noise mask overlapping the target location, the performance decreased in both tasks. Accordingly, it was argued that the target was vulnerable to masking only when the task required feature binding. If this argument is the case, it follows that the same discriminability was maintained, because our task did not require any binding, but it could be performed as long as the orientation of a Gabor patch was discriminated.

In contrast, Goodhew et al. (2015) found that the performance in orientation discrimination for a Gabor target decreased only when a mask with an orientation similar to that of the target was used, arguing that the feature similarity between the target and mask rather than the complexity of the target was a determinant of the discriminability reduction during common-onset masking. This argument is in good agreement with the object updating theory (e.g., Goodhew, 2017, Lleras & Moore, 2003; Pilling & Gellatly, 2010) and suggests that not only the difference in color (e.g., Moore & Lleras, 2005) and the difference in luminance polarity (Luiga & Bachmann, 2008) but also the dissimilarity of orientations between the target and mask facilitate object individuation, thereby releasing the target from masking. If this argument is the case, another possible reason why discriminability was not reduced might be that the Gabor target and dot mask are not very similar to each other. The above reasons may be complementary rather than mutually exclusive.

Although there is no evidence of discriminability reduction, we argue that the general operations of backward masking in the situation of the conventional literature and ours are consistent with each other, as discussed in the following sections.

### Backward masking disrupts contextual modulation

The finding that backward masking disrupted the process of contextual modulation is consistent with physiological findings. The responses of macaque V1 neurons to the stimuli inside their classical receptive fields are modulated later, depending on whether the stimuli belong to the figure or ground with respect to other stimuli outside the receptive fields (Lamme, 1995; Zipser, Lamme, & Schiller, 1996). Lamme, Zipser, and Spekreijse (2002) recorded the responses of macaque V1 neurons to orientation-defined figure-ground stimuli followed by a pattern mask with various SOAs. At intermediate SOAs, the figure-ground modulation was weakened, with the orientation selectivity of the neurons remaining intact. Furthermore, the figure detectability quantified by a saccade task was correlated with the strength of figure-ground modulation more than orientation selectivity. Thus, it was concluded that figure detectability was lowered because backward masking disrupted contextual modulation. Similarly, a human electroencephalogram study demonstrated that backward masking selectively disrupted later activities in early visual areas possibly involved in reentrant activities, while leaving intact earlier activities in higher-tier areas possibly involved in feedforward activities (Fahrenfort, Scholte, & Lamme, 2007).

However, to our knowledge, we obtained the first psychophysical evidence that backward masking could disrupt contextual modulation, and this disruption could result in altered appearance—a completely different phenomenon from the detectability reduction demonstrated by the physiological studies mentioned above.

### Backward masking alters orientation appearance

According to de Gardelle, Kouider, and Sackur (2010), one type of oblique illusion in which the orientation of an oblique target is overestimated (see Smith, 1962) covaries with the subjective visibility levels of the target. More specifically, the strength of illusion increased with visibility up to some level but then decreased as visibility was further increased, indicating a nonmonotonic change in orientation appearance, which was attributed to the difference in the extent of cognitive resource allocation.

Similar to this type of oblique illusion, orientation repulsion is also considered to involve multiple levels of visual processing hierarchy, possibly including the level where cognitive modulation profoundly takes place (Clifford & Harris, 2005; Wenderoth & Johnstone, 1987). As discriminability was not altered by common-onset masking in our experiments, cognitive resources might have been allocated to the same extent in the simultaneous-offset and delayed-offset conditions; thus, the cognition-based explanation by de Gardelle et al. (2010) is not viable for reducing repulsion, for which a process essentially parallel to that involved in the evolution of discriminability must be considered.

### Extended object updating theory for common-onset masking

Previous studies have proposed several theories and computational models that account for the discriminability reduction in common-onset masking. Here we discuss the extent to which our findings are consistent with these studies and how they can be extended. First, Põder's (2013) model, assuming two-stage noise addition processes, may pertain to our concern that the physical arrangement of the four-dot mask might have modified the target's apparent orientation in Experiment 1, if the model is extended to include a noise addition process in the orientation dimension. As mentioned earlier, we were concerned that the four-dot mask constituting an upright square could have served as a vertical reference. Even if observers did not intentionally use the vertical orientation as a reference, it was possible that a noise addition process in the orientation dimension automatically compromised the repulsion. However, the reduction of repulsion was replicated in two control experiments as well as in Experiment 2, in which the constellation of verticality was minimized; thus, it is unlikely that noise addition was responsible for the reduction of orientation repulsion. For the same reason, the CMOS implementing simple temporal integration and attentional gating mechanisms (Di Lollo et al., 2000; see also Põder, 2013) is not sufficient.

Second, two theories assuming object-level interferences between the target and mask are considered: object substitution theory (e.g., Di Lollo et al., 2000; Kahan & Lichtman, 2006; Pilling et al., 2019) and object updating theory (e.g., Goodhew, 2017; Lleras & Moore, 2003; Pilling & Gellatly, 2010). As the differences between these theories are subtle and the present study did not aim to differentiate them, we tentatively consider the object updating theory as a working hypothesis because it imposes looser constraints on the mechanism of common-onset masking compared to the object substitution theory. However, the involvement of reentrant activities in common-onset masking assumed by the object substitution theory is not inconsistent with our suggestion that the internal representation of the target orientation evolves over time. In addition, the assumption of the object substitution theory— that focusing attention on the target location reduces the common-onset masking effect—is neither supported nor refuted because we did not manipulate spatial attention between conditions.

In any case, to interpret our findings that common-onset masking altered not the discriminability but the suprathreshold appearance of the target, it is necessary to extend the notion of object updating to a temporally evolving process of suprathreshold appearance as follows. Here we tentatively focus on the representational dynamics at V1, the first stage of explicit orientation representations. First, the direct responses to the target onset initiate the formation of a representation of target orientation faithful to the light distribution on the retina. If the target orientation is vertical, for example, a population of orientation-selective V1 neurons initially represents the vertical orientation. This formation process is not disrupted by backward masking (see Enns, 2004). Next, since the initial internal representation is subliminal due to its weakness and instability, the signal-to-noise ratio of the target must be increased by some time-consuming process, perhaps involved in reentrant activities between V1 and higher-order visual areas, as assumed in the object substitution theory (Di Lollo et al., 2000; see also Lamme & Roelfsema, 2000). Parallel to this, contextual modulation from inducers coming up later causes the internal representation to evolve slowly and be repelled from the inducer orientation. The divisive normalization model (Goddard et al., 2008; Schwartz et al., 2009) dictates that, if the target orientation is vertical and the inducer orientation is 20° (clockwise from the vertical), for example, the responses of V1 neurons that prefer the target location and 20° orientation are selectively normalized within a certain period; this tuned normalization process renders the orientation represented at a population level tilted counterclockwise from the vertical. The responses of V1 neurons are also known to be sluggishly modulated by contextual stimuli outside their classical receptive fields through feedback from higher-order visual areas (see Lamme, 1995; Zipser et al., 1996). The former signal-enhancement process involved in discriminability evolution is completed earlier because of two possible reasons: the discrimination of target orientation may be performed without a time-consuming feature integration process (see Bouvier & Treisman, 2010); and object individuation between the target and mask may be easy and fast because of their dissimilarity (see Goodhew et al., 2015). In any case, our stimulus configuration, including the use of a Gabor target that could be discriminable only based on the orientation information and looked dissimilar to the four-dot mask, might contribute to the fast evolution of discriminability. Conversely, if a target was more complex and looked more similar to a mask, it might take more time to render a visual content discriminable and thus become affected more severely by the mask. Here we assume that discriminability evolution takes less time than appearance evolution involving the latter contextual modulation process. In the simultaneous-offset condition, both the discriminability and appearance evolution are completed because there is no interruption by backward masking; thus, the maximal repulsion is reported. In contrast, in the delayed-offset condition, the establishment of object correspondence between the target and mask triggers the process of object updating (see e.g., Goodhew, 2017). Object updating compulsorily terminates appearance evolution, rendering contextual modulation weaker. If backward masking interrupts the reentrant activities between V1 and higher-order visual areas (Boehler, Schoenfeld, Heinze, & Hopf, 2008; Di Lollo et al., 2000; Lamme et al., 2002), the termination of evolution might occur in visual areas as early as V1. However, the target is sufficiently discriminable because discriminability evolution is completed prior to object updating. In other words, the target orientation has already been represented as a preconscious form beyond a subliminal form by the time the updating occurs. Consequently, the premature representation is consciously accessed; thus, reduced repulsion is reported. We should note that Clifford and Harris (2005) reported a superficially related finding that orientation repulsion was reduced when not a target, but an inducer was masked and gone unnoticed. Despite their intriguing claim that orientation repulsion involves a multilevel processing hierarchy, their manipulation of masking did not reveal a temporally evolving process underlying repulsion and masking, whereas we did for the first time by masking the target.

Although here we focus on orientation representation, the above-mentioned process of temporal evolution can theoretically be applied to other domains of internal representation underlying various conscious phenomena. In addition, backward masking might be redefined case-by-case as the terminations of such distinct temporal evolutions.

The extended object updating process proposed above might be similar to the temporal trimming process suggested by Harrison, Rajsic, and Wilson (2017), who examined the common-onset masking effect on a long-lasting target. In their temporal order judgment task, the duration of a target actually presented for 200 ms was perceived as 11 ms shorter in the delayed-offset condition compared to the simultaneous-offset condition. When observers were asked to indicate the last perceived tilt of an isosceles triangle target that had been revolving around the fixation point while rotating until it disappeared, they tended to report an approximately 2 ms earlier tilt in the delayed-offset condition compared to the simultaneous-offset condition. Harrison et al. (2017) termed this effect temporal trimming, suggesting that common-onset masking compulsorily shuts down the temporal window for sampling the target and forming its conscious representation.

Under the assumption that the reduction of orientation repulsion in our study was also caused by temporal trimming, we estimated the duration of target representation trimmed by common-onset masking in Experiment 2. The effects of temporal mismatch on the proportion opposite data had a significant quadratic trend (see Results and discussion in Experiment 2); therefore, we fitted each of the data in the simultaneous-offset and delayed-offset conditions with a quadratic function. The estimated temporal mismatch at the intersection of the best-fit curve and the horizontal line indicating the unbiased response (i.e., 50%) was 106 ms in the simultaneous-offset condition and 97 ms in the delayed-offset condition. We regarded their difference, 9 ms, as the trimmed duration in our situation because this value amounts to the difference in temporal range within which inducers could modulate the internal representation of the target orientation. Thus, the estimated trimmed duration in our study was in the same range as that in Harrison et al.'s (2017) study.

## Conclusions

We examined the strength of the orientation repulsion and its time course during common-onset masking and found that masking reduced the repulsion only when inducers were presented together with or after the target, while it did not hamper orientation discriminability. Therefore, we conclude that the internal processes required for discriminability take less time, but that contextual modulation of the suprathreshold appearance in orientation repulsion takes a longer time. In this case, masking compulsorily terminates the temporal evolution of appearance and allows a premature representation in the middle of temporal evolution to arise in one's conscious awareness. These temporal evolutions of internal representation behind discriminability and appearance are broadly consistent with the previous notion of object updating.

## Supplementary Material

Supplement 1
