# Supplementary material for: Common-onset masking terminates the temporal evolution of orientation repulsion
Source: J Vis. 2021 Aug 3;21(8):5. doi: 10.1167/jov.21.8.5 (PMC8340666; doi:10.1167/jov.21.8.5)
Supplement: Supplement 1 [file jovi-21-8-5_s001.pdf]

Supplementary materials for: Common-onset masking terminates the temporal evolution of orientation repulsion

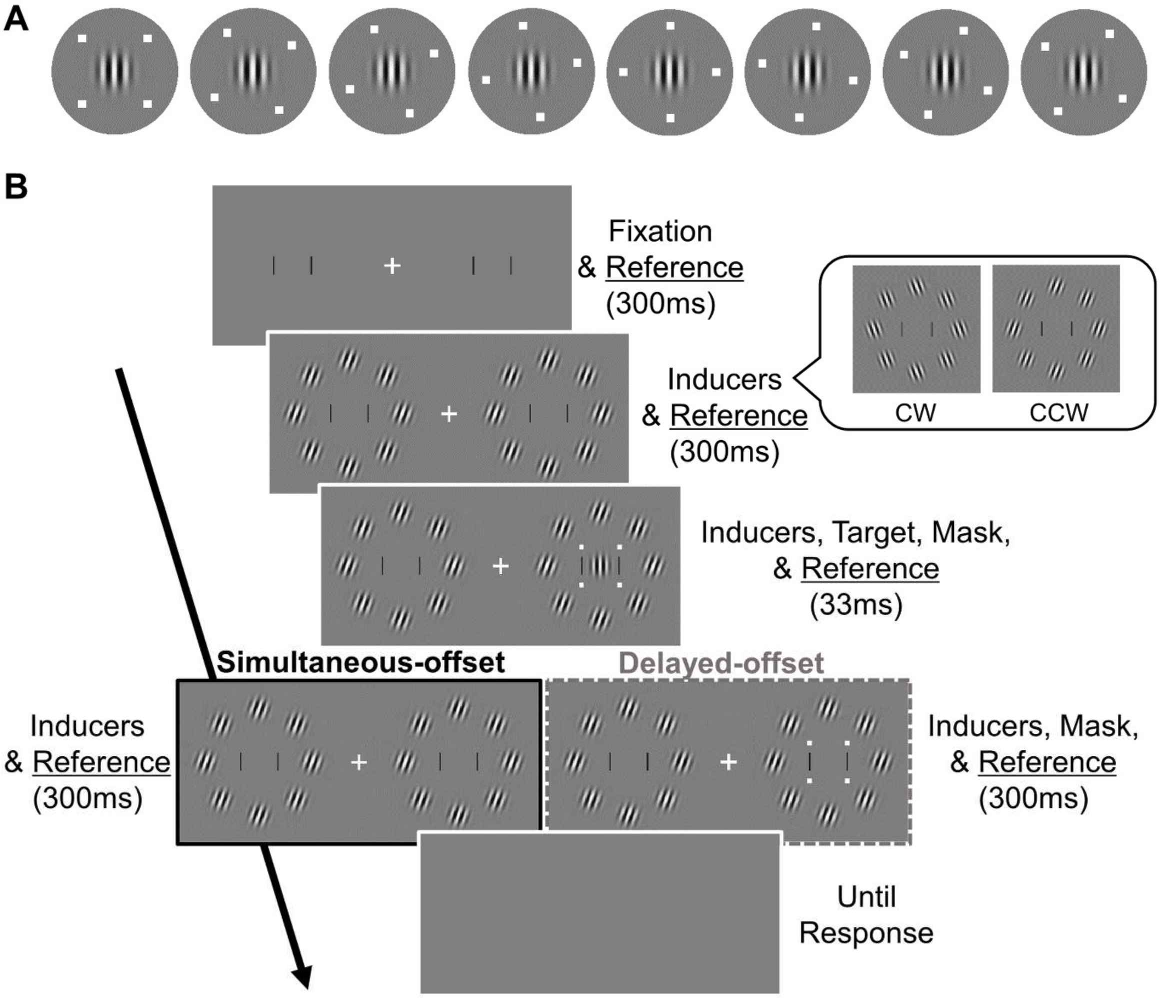

Figure S1. Methods of the two control experiments. (A) Eight orientations of a virtual square composed of four dots used in Control Experiment A (0, 11, 23, 34, 45, 56, 68, and 79 deg). The procedure was the same as in Experiment 1 except that the mask orientation was chosen in a random order. (B) Time course of each trial in Control Experiment B. The procedure was the same as in Experiment 1, except that two black bars were presented between the possible locations of the dots at each side as vertical references, and that we omitted the baseline condition in which no repulsion was expected to occur.

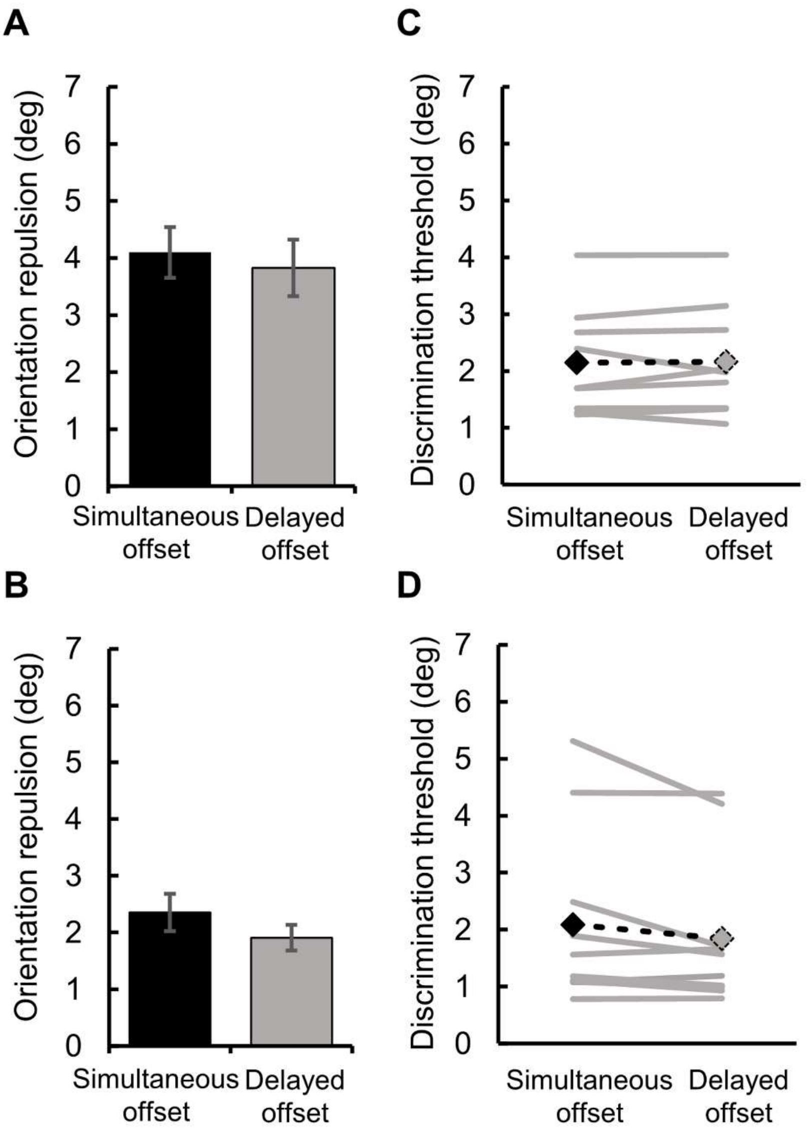

Figure S2. Results of the two control experiments. (A) Interobserver mean of orientation repulsion in Control Experiment A. (B) Interobserver mean of orientation repulsion in Control Experiment B. The black and gray bars indicate the simultaneous-offset and delayed-offset conditions, respectively. The error bars are the standard errors of means. (C) Discrimination threshold for orientation in Control Experiment A. (D) Discrimination threshold for orientation in Control Experiment B. The solid gray lines indicate individual data and the dashed black line indicates the interobserver mean.
